# Periscope

**Published:** 1832-07-01

**Authors:** 


### Periscope;

OR,

### CIRCUMSPECTIVE REVIEW.

"Ore trahit quodcunque potest, atque addit acervo."

### I.

### Injection of Water into the Veins in a Case of Hydrophobia.*

A boy, of the name of Ménard, was brought to the Hôtel Dieu with all the signs of confirmed hydrophobia. The paroxysms occurred, as usual, at intervals, and were very severe; the pulse was very frequent, without force; the respiration not difficult; the eyes wandering; the mouth frothing; the countenance inexpressibly anxious. Large bleedings had been practised without any benefit, and at 9 o'clock, M. Sanson proceeded to inject warm water into the veins of the patient, as M. Magendie had previously done in the case which, at the time, excited so much attention in Europe.

The left radial vein was chosen for the operation, and an assistant having fixed it by pressure in the middle of the arm, and rendered it dilated below the point compressed, an incision, an inch and a half in length, was made on it, parallel to its long axis. It was then seized with a pair of forceps, and a longitudinal incision, two lines in length, made into it. The assistant's finger restrained the hæmorrhage, when the operator, raising the upper end of the vessel with a pair of dissecting forceps, introduced into the aperture the pipe of a very delicate syringe, containing nearly eight ounces of distilled water, at the temperature of 25 to 28 degrees. On attempting to throw in the fluid, it was found to be thrown into the cellular membrane of the limb. The pipe was, therefore, withdrawn, the syringe re-filled, and re-applied with more success; four ounces of fluid being injected with facility. The instrument was then removed, and the lips of the wound brought together as usual. During the operation, the young patient was much disturbed, but less so than before, and immediately after its completion he fell into a state of calm, which continued till his death, interrupted only by some slighter convulsions, which followed a bleeding at 10 o'clock. From this moment the pulse became more frequent and weak, respiration more difficult, deglutition almost impossible. In this state, after a few minutes' quiet, he expired.

This case can hardly be looked on as conclusive against the powers of aqueous injections into the veins in cases of hydrophobia, and yet it will have its weight in preventing enthusiastic persons from leaning much on M. Magendie's proposal. That proposal has so little, in the way of reasoning, to recommend it to serious attention, or, at least, to excite much expectation of success, that every practical failure in its application contributes most heavily to sink it. By the way, we may here mention a circumstance which is remarkable in itself, but more curious, as evincing some of those national characteristics peculiar to the French nation. When we have described the circumstances, our readers will perceive that the characteristics to which we more especially allude, are that enthusiasm, and almost reckless daring, which have rendered the terms of Frenchman and courage almost synonymous. We willingly pay this tribute to those, whom absurd and ignorant prejudice has still styled natural enemies, when

* Bulletin Général. Paris, 1831.

they should have been embraced as natural neighbours.

It appears, then, that the boy whose case forms the subject of the present notice, whilst suffering in one of the hydrophobic paroxysms, seized a piece of cloth, and, thrusting it into his mouth, was in imminent danger of suffocation. M. Caillard, resident physician of the Hôtel Dieu, boldly put his hand into what most men would consider an awkward situation, extricated the boy from his present danger, and reserved him, cruelly perhaps, for equally certain, though more tedious destruction. In executing this object, the boy inflicted a deep bite on the thumb of the right hand; the wound was immediately cauterized by the hot iron. This gentleman seems to pay no regard to the wounds inflicted by the rabid of his own species. Some little time ago, he overpowered two hydrophobic patients whom none would venture to touch, and received a bite in the finger from one, who had made his escape over the roof of the Hôtel Dieu, but was followed and brought back by M. Caillard. The latter experienced no bad consequences from that injury, and, we trust, will suffer none from the more recent. Another circumstance is mentioned, which we cannot pass without remark. An amateur of experiments had inoculated ten dogs with the hydrophobic virus, and having made them as mad as could be wished, became naturally desirous of getting rid of them. Now most men would have despatched such animals as speedily, or, at all events, as securely as possible; but this philosopher, disdaining such commonplace precautions, ties them up loosely in a bag, and commissions a poor wretch to throw them into the Seine. The man, little dreaming what a cargo he is carrying, stops at an auberge to take some wine, and eases himself for a minute of his load. The dogs escape from the sack, and the *experiment* is concluded by sixty-four persons being cauterized, at the Hôtel Dieu, for wounds inflicted by them, and ten, actually ten, *dying* in that institution of hydrophobia! Has the law in France no means of punishing such atrocious carelessness, or rather callousness? We cannot refrain from observing, that the proportion of ten deaths is very large, and not particularly creditable to the cauterizing practice. Two or three persons are annually received into the Hôtel Dieu, affected with hydrophobia; they most commonly die at the end of from twenty-four to forty-eight hours.

---

### II.

### ARTERITIS.

INFLAMMATION of the arteries may be said to be little understood, and morbid anatomy being much cultivated at present, whilst the study of symptoms and of pathology, in its comprehensive sense, is by no means neglected, some powerful reason must exist for our ignorance upon the subject. Perhaps attention has hitherto been too little directed to it, and the investigation has not been followed up with that unity of purpose and perseverance, which are requisite for the elucidation of any thing at all enveloped in obscurity. But much, and probably the greater difficulty, must be attributed to circumstances connected with the question itself, to the fact, that the arterial tunics are not prone to extensive acute inflammation, and that the changes produced in them by old age are with difficulty distinguished from the results of chronic inflammation. In our review of Mr. Guthrie's able work, we have touched on this disputed point; but, in the present state of our knowledge, we would be far from wishing to maintain opinions pertinaciously, and no one should decide upon grounds not perfectly supported and consolidated by evidence. In the second number of the Cyclopædia of Practical Medicine, there is an article on Arteritis from the pen of Dr. Hope, of which we shall take a short notice.

On acute arteritis we have little that is satisfactory; a compiler cannot collect more than is known, or rather than has been written; and if knowledge is scant, a compilation cannot increase it. The redness of the inner tunic or tunics of an artery, which is often thought and said by ill-informed persons to be inflammatory, but which really is not so, is distinguished from the vascularity which has better claims to be considered the result of inflammation. Were we to attempt to define, we should say that the non-inflammatory redness has the properties of a *stain*, the inflammatory those of vascular *injection*. And observing men will find that the cases of the latter are passing few, at least we have not witnessed many. We recommend a rational scepticism in these investigations. The most unequivocal symbol of inflammation is lymph, and if lymph be present on the inner tunic of the artery, we may conclude that arteritis has really existed. We may conclude so if lymph is really present, but our readers should be aware that they are exposed to a fallacy. The fibrine of the blood when adherent to the sides of an artery or vein, has many of the appearances of coagulated lymph, nay, is with great difficulty distinguished from it. In persons dying after severe inflammatory affections, in which great depletion has been used—for instance, after pericarditis—or from long-continued chronic disease, by which the vital powers are brought low, and for some time maintained so, we occasionally observe a very curious condition of the veins of one of the lower extremities. As it seems to us to illustrate the present subject, we may here allude to it. For some days then before death one of the lower extremities swells considerably, and becomes œdematous, sometimes with great pain in the limb affected, sometimes with little or none; occasionally this state attracts no attention during life. On inspection after death, the femoral, and perhaps the iliac, vein is found completely obstructed, and the same is the case to a greater or lesser degree with their branches. The veins appear large and knotted, but the coats present externally no redness, at all events no injection. On opening the vessel, its interior is found filled with a darkish brown substance, which has partly the appearance of coagulum, partly that of lymph. It is disposed to be laminated, adherent to the sides of the vessel, in its centre softened, like those coagula in the heart which are semifluid in their interior, and are incorrectly said to contain pus.

Now we have seen these cases viewed as instances of phlebitis. We are by no means satisfied that they are so, and for these reasons. In undisputed examples of phlebitis there is more unequivocal coagulated lymph, less coagulum, and always more or less thickening of the coats, or deposition of matter so organized that it is difficult to separate it from the venous tunics. Besides, in acute phlebitis, there are usually geral symptoms of a typhoid or peculiar character, which, so far as we have seen, are absent in these cases. The circumstances attending the latter, resemble those accompanying the altered coagula in the heart;—a long and debilitating malady, by which we may suppose the powers of the circulating system weakened, and the disposition of the blood to coagulum increased—the act of dying generally protracted and the struggle tedious—the occasional absence of all local pain or uneasiness, and the deficiency of the general symptoms of phlebitis, so obscure to those who are not, and so characteristic to those who are acquainted with them. Be this as it may, the appearance of the matter in the vein—blood or lymph—is in all respects analogous to that of the pseudo-abscesses in the heart, which were once like these considered inflammatory, but are at present generally acknowledged to be merely altered coagula.

We think that this digression will shew that the presence of lymph in the artery is not so easily determined as it might seem to be; and that conclusions must not be jumped at too hastily, on observing the adhesion of "plastic pseudo-membranous lymph" to its sides. Another consequence of inflammation is ulceration, and the latter may be thought very frequent. Fissures of the ulcerated character are indeed very frequent, along with the deposites between and in the coats, more particularly with the scales of calcareous matter. But independent of these they are extremely rare; independent of the presence of morbid alterations in the coats, we never saw an instance of them. "Small pustules filled with pus sometimes, though rarely, present themselves under the internal membrane of the aorta, and burst into its cavity." Laennec thinks very reasonably that they originate in the cellular tissue of the vessel. Here then we see how uncommon the unequivocal results of inflammation are, how equivocal the common proofs of it.

Arteries are undoubtedly susceptible of inflammation limited in extent, and in degree. They exhibit the adhesive inflammation after wounds, injuries, ligatures, in the neighbourhood of suppuration, ulceration, sloughing. Arteries also ulcerate with the soft parts, and they slough with them. But it may be observed that these morbid processes are not in themselves dangerous, but rather sanatory—that the vessels affected are usually the vessels of the part implicated, and no others—and that coagulation of the blood in the vessel is the ordinary precursor both of its adhesion, ulceration, or death. These, though examples of inflammation in arteries, can hardly be viewed as examples of acute arteritis, taken in its broad and fair signification.

But the gravest cases of arteritis are to be noticed, and we think they deserve more than the paragraph which Dr. Hope has bestowed upon them. It is true that they are witnessed more frequently by the surgeon than by the physician, and this offers an example of the inconveniences which arise from articles of this description being confided to one individual. With a show of exactness and completeness they are deficient in both, for no physician can be properly acquainted with portions of surgical knowledge which are rather the possession of a few experienced men, than generally given to the world in books.

There is good reason to believe that however limited and free from danger inflammation of arteries usually is, cases do occur of a more formidable character. The inflammation spreads extensively and towards the heart, is said to assume the erysipelatous form, and too commonly proves fatal to the patient. The following quotation is taken from Mr. Guthrie's able work on the diseases of arteries; it is short and to the purpose.

"I have alluded to one case of this kind in my work on Gun-shot Wounds, page 96, third edition. The patient died suddenly and unexpectedly, the limb was greatly swelled and gorged with blood, the femoral artery, when opened, appeared more vascular than is commonly observed, its internal membrane was very red, and easily separated from the middle coat, and the fluid which lubricated its surface was of a more serous nature than usual, and greater in quantity. The other two cases I did not see until death was impending. In each, the right leg and thigh were greatly swelled and œdematous; the skin was of a pale dead white colour; the countenance was extremely anxious and bedewed with sweat; the pulse 140 and weak; the patient sensible, and expressing hopes of recovery. In both cases the arteries showed the same character of disease, which extended upwards as high as the diaphragm. I gave a portion of the femoral artery taken from one of these cases of disease to Mr. Brookes, who considered it to be one of the finest specimens of arteritis he had ever seen. The symptoms which mark this state of disease, when distinguishable, and several cases are recorded where the appearances described have been noted, are a very quick pulse, a rapid deterioration of the state of the patient, and degeneration into irritative fever, with low delirium, followed by death.

In the three instances which I have mentioned, the erysipelatous inflammation of the arteries was evidently caused by continuity of parts with those already inflamed; and in all of them the inflammation was a deep-seated disease, situated in the muscles and internal structure of the thigh, the skin being apparently unaffected."

It will be observed that the foregoing extract by no means conveys an unexceptionable idea of either the morbid characters, or nature, or progress of acute arteritis, if such it was. It is probable that arteries should be subjected, like other tissues, to acute and severe inflammation, and it is more than probable that, if so affected, the result would be fatal, or at least problematical. Yet we think our readers will agree with us, that hitherto our information is very scanty, and that cautious men must hesitate ere they subscribe to this or that position respecting the symptoms, nay, existence of the disease. One of the least dubious cases of arterial inflammation of which we have heard, was related by Mr. Brodie, in his surgical lectures.

*Case.* A man was walking on a hot day in the fields of Brompton, when he was seized with violent pains in the limbs, one of which was affected more severely than the other. The foot of that side mortified, and became dry, hard, and horny; in short, was attacked with dry gangrene. The mortification reached the middle of the thigh, where, there being much flesh, the parts became moist and putrid, and the mortification was arrested; but the man died. On dissection, both artery and vein were found inflamed from the middle of the iliacs to the middle of the thigh; there was an effusion of coagulable lymph in the interior, and there was lymph also in the cellular membrane.

Another case of a similar description was also mentioned by Mr. Brodie, but the patient having recovered, the presence of lymph in the artery is not so certain. The case is interesting, and we shall mention it.

*Case.* A young woman was seized with pain in the groin, extending downwards in the course of the femoral artery, and upwards into the abdomen. The foot became affected with dry gangrene, but the mortification stopped in the middle of the thigh, separation down to the bone took place, Mr. Brodie sawed through it, made a good stump, and the young woman did well.

In the notes that we possess of the lecture from which these cases are extracted, Mr. Brodie is made to remark, that "he has seen a finger attacked in the same manner, and mortify in a single night. Saviard amputated in one of these cases, and there was no vessel to be tied, for all were choked up."

These cases differ from the mortification of old persons, in which the coats of the arteries are rigid, diseased, their cavities diminished, the heart perhaps unsound, the circulation certainly very feeble. In such, sudden coagulation of the blood in a vessel of an extremity, and consequent gangrene, is not uncommon.

We suppose that, from what has been said of acute arteritis, we may save ourselves any trouble respecting the symptoms and diagnosis. Of what use is it but to mislead, to affect a knowledge which we really have not, and to lay down the symptoms of the disease, of the very existence of which we are scarcely certain—symptoms which are in some measure speculative, in some measure false—symptoms which belong to other affections, even if they belong, as is not certain, to this! The same thing may be said of the treatment; the hare must be caught before it can be cooked.

As, in our review of Mr. Guthrie's work, we entered on the consideration of chronic arteritis, we cannot resume it here. If, in this article, we have rather discouraged pretensions to a knowledge of the nature of arteritis, it is not to repress, but to stimulate enquiries—to make us dissatisfied with a state of half information, half ignorance, in order that we may eagerly endeavour to attain positive knowledge. And we think it is more philosophical to sin on this side, than on that of confidence and easy faith. We cannot conclude without expressing our opinion, that Dr. Hope has executed his task with zeal and learning.

### III.

### MESSRS. GREEN AND S. COOPER ON FRACTURE OF THE CERVIX FEMORIS WITHIN THE CAPSULE.*

THE disputes which have been agitating the surgical world for some years respecting the possibility of union of fractures of the neck of the femur within the capsule appear to be dying away. Both parties seem weary of the contest, and each is willing to relax a little from his ultra opposition to the other. On the whole, however, the union-party have rather the best of the argument, at least of the facts, for they have managed to bring forward some which cannot be readily disproved. Sir Astley Cooper, however, never asserted that union in these fractures was impossible, and the very few unexceptionable examples of it adduced by his opponents, may be said to be proofs of the general soundness of Sir Astley's opinion. Mr. Green's sentiments on this subject are as follows :—

"Now, gentlemen, whatever be the causes of it, the fact is, at all events, clear, that universally, I may say, fractures of the neck of the thigh bone within the capsule, are deemed to be incapable of ossific union. Dr. Colles, of Dublin, who has paid great attention to this question, has placed on record the appearances which presented themselves in dissection, in a considerable number of fractures of the neck of the femur. Sometimes union was formed by ligamentous bands, longer or shorter. Here is a specimen of a similar union, where you may observe the ligamentous bands to be very short, indeed, so much so, as to be capable of maintaining a permanent attachment between the broken parts. Next, Dr. Colles found instances in which the neck of the bone was absorbed without any union. He found, however, in the lower portion of the fractured bone, deposits, forming a case — and sometimes acting like a splint in supporting the neck. Thirdly, he says that the broken surfaces present patches of ivory-like deposits, that they move upon one another; and that thus, in some measure, support is maintained.

Such are the general results which have been arrived at upon this important point of practice. For my own part, I cannot help believing that bony union, in such cases as we are now considering, is perfectly possible. I saw some time ago, and minutely inspected, a specimen of bony union of the neck of a femur, sent to me by Mr. Chorley, of Leeds, and which I have no doubt was a fracture within the capsular ligament. Complete union was established, and it is only just to mention, that Mr. Chorley so considered it at the time. I have seen another specimen of the same process, and cases of the kind are mentioned as having occurred in the practice of Dr. Bruedo and of Mr. Field. In short, there can be no doubt of the possibility of bony union in fractures of the neck within the capsule, although I am ready to admit that such cases are extremely rare.

Now, gentlemen, seeing how seldom in this class of fractures, union by ossific matter takes place, you may be inclined to ask, why not give up the hope of such complete union at once, and direct all your treatment to the facilitating of that sort of union which most commonly takes place in such accidents? The answer is, that since bony union is possible, and since close ligamentous union will answer the intended purpose, it is our duty to afford the patient the best chances of regaining the most useful state of the limb. Now, this we effect by striving to produce ossific union. But I really cannot but think, after all, that the want of bony union in all these cases, is to be attributed principally to the imperfection of the mechanical treatment which is applied to the limb. For, in the first place, as to the obstacle, of the want of apposition, I am sure I could shew you that the muscles by which this impediment is produced may be completely controlled. Then, as to the deficiency of vascular supply in the head of the bone, and of consequent organic power to generate ossific mat-

* London Med. and Surg. Journ. No. I.

ter, I can only observe, that you have deposits—if not of bone—certainly of ivory patches, sufficient to satisfy you that there exists in the separated head a power to produce the substance necessary for a complete union. But on this part of the lecture I shall not say more until I have spoken to you of fractures external to the capsule."

On the latter Mr. G. says nothing new. The treatment recommended by Mr. G. for fractures internal and external to the capsule is the apparatus of Mr. Amesbury. Mr. Cooper may be said to be a degree or two higher on the scale of the unionists than Mr. Green. Perhaps he may be considered as evincing rather too much facility in the admission of facts, but the lecture is characterized by impartiality and judgment.

"In whatever manner," says Mr. Cooper, "we may reason upon this matter, facts must remain unaffected by our particular notions concerning the manner of their production. Many cases of bony union of transverse fractures of the neck of the femur within the capsular ligament are now upon record, and their reality is confirmed by dissection and the preservation of the parts. I know of about ten examples myself. You may read some of the particulars, of not less than four, in *Amesbury's Treatise on Fractures* It is from one of these examples that the specimen, which I now produce, was taken by my friend Mr. Langstaff, who has been so kind as to lend me the preparation and accompanying drawings. If you examine them, you will see, that though the fracture has been completely within the capsular ligament, entirely through the neck, which has been absorbed, a firm bony union has taken place. In the line of fracture, in a kind of groove, which you may observe towards its centre, there was indeed a ligamentous substance, yet the bony connexion elsewhere is perfect and solid.

Another equally satisfactory proof of bony union is exemplified in the case of Dr. James, who fractured the neck of the thigh-bone by a fall from his horse in the South of France, and was attended by Dr. Brulatour, of Bordeaux. Dr. James having died of some pulmonary complaint seven months after the accident, the surgeon, whom I have mentioned, had an opportunity of dissecting the hip-joint, and of ascertaining the state of the fractured bone. Here are drawings of it, from which you will perceive that the case was a transverse fracture entirely within the capsule, that the neck is absorbed, and that the opposite ends of the fracture are even more completely consolidated by bony matter, than in the interesting case published by Mr. Langstaff, in the *Medical and Chirurgical Transactions.* I might quote other cases in proof of the occasional accomplishment of bony union; but these will perhaps suffice. We have no specimen of bony union of a transverse fracture of the neck of the femur entirely within the capsule in the museum of this University. All the cases are either not united at all, or united by ligamentous bands. But we possess the curious preparation already specified, in which the fragments of the head of the bone are consolidated, though the neck itself remains ununited. In the greater number of such cases, the accident leads to the absorption of the whole, or greater part of the neck, and whatever strength the joint regains, is owing to ligamentous bands, which generally extend from the inside of the capsule to each fragment, but sometimes from one fragment to the other. In many instances, the capsular ligament is also considerably thickened.

In the specimen which I now hold up, you see a fracture of the neck of the femur, within the capsule: the head of the bone is broken off at the commencement of the neck: these were the appearances seven weeks after the accident: you may remark, that there is no attempt at union.

In another example, which I shew you, the neck of the femur was broken, the head is much diminished and displaced, and nearly the whole of the cervix absorbed.

Here we have another preparation, in which the fractured neck of the bone is united by ligament.

In another specimen upon the table, you will see a fracture of the neck of the bone, where osseous union has taken place. On looking at its posterior surface, it is clear that the fracture extended within the capsular ligament; on looking at its fore-part, this is doubtful. The head of the bone is depressed, and the limb consequently shortened. These fractures, extending beyond the limit of the synovial membrane, as I have explained, commonly unite by bone. Here, however, is another preparation, in which the head of the femur is broken, and, though the fracture extends through the neck to the outside of the capsule, the uniting substance is ligamentous. But, as only three weeks had transpired from the time of the accident, when the patient, an aged female, died, it seems to me probable, that if she had lived longer, the union would have been of an osseous nature.

Gentlemen, I now request your attention to two facts of practical importance, brought before us by the foregoing views:

The first is, that we cannot always be positive about the fracture being entirely within the capsular ligament.

The second is, that the majority of cases, when they are of this kind, admit of union by ligament, and a few by bone; while all others reaching beyond the limit of the synovial membrane towards the trochanter, having more or less of a longitudinal direction, generally unite by bone, as well as common fractures."

Mr. Cooper considers it manifest that our duty, as surgeons, should always be to aim at bringing about union, by whatever substance it may be accomplished. He enjoins, whatever apparatus be employed, the quiet maintenance of the limb in a certain posture, without any attempt at pressure directly over the hip. Mr. C. does not appear to give a decided preference to any particular apparatus.

---

### IV.

### Hospital and Clinical Reports.

### I. SINGULAR TUMOUR FROM THE UTERUS—MR. LAWRENCE AND DR. CONQUEST AT FAULT.

[St. Bartholomew's Hospital.]

OUR new contemporary, the London Medical and Surgical Journal, for as a weekly contemporary it is new, although, as a less frequent coadjutor, it is more antique, our new contemporary, then, has published a remarkable case which occurred to Mr. Lawrence and Dr. Conquest, and appears to have sorely puzzled these not inexperienced doctors. Ars longa, vita brevis, experientia fallax, said Hippocrates, and modern surgery must share in this instance a little of the opprobrium of ancient physic. But to the point. The case is headed "Caution to young Practitioners," and comes in the shape of a clinical confession, or homily, from Mr. Lawrence. We must give it entire, for it is brief and to the purpose.

"GENTLEMEN,

In the course of your practice you will often have occasion to exercise great caution and vigilance in ascertaining the nature of the complaints with which the patients who consult you represent themselves to be affected. You will have to guard against attempts at deception; and you will do well not to trust at all times to the explanations which you may hear. I am about to describe to you an instructive example, which particalarly illustrates this precept. A young woman was recently received into this hospital, and her disease was said to be gonorrhœa. She was a patient in Faith's Ward. After a few days the discharge from the vagina was observed to be of a colour very different from that usually seen in the gonorrhœal fluid: it was dark-coloured, and almost intolerably fetid. A suspicion began to be entertained, that the disorder was of a more serious character than was at first supposed; an examination of the uterus therefore became necessary. Upon carrying the finger to the os uteri, I felt it somewhat hardened; and from it protruded a substance extremely soft and elastic, which bore no marks of polypus, or indeed of any other uterine excrescence with which I am acquainted. It was elongated—not very large, and appeared to be attached to the internal surface of the uterus by a very thin peduncle. I found no appearance of disease in the uterus, and I concluded that this growth could have been easily removed. In the meantime the chloride lotion was used, and the discharge was deprived of its foul odour at least. This young woman had been a companion to some gentleman, but her complaint, as she herself acknowledged, was a complete bar to that fruition which she was led to expect. I determined upon extirpating the tumour; but before doing so, I thought it prudent to request of Dr. Conquest, whose peculiar experience might have enabled him to form a better judgment than I could of the young woman's disease, to visit and examine her. He accordingly paid her a visit. He proceeded to an examination; but he could not declare the nature of the tumour—although he said that he was sure he could remove it without much trouble. He was urged, however, to do so; and after examining a second time, he pulled away the substance which proved to be nothing more or less, gentlemen, than a piece of——sponge! (A laugh.) It had been there for a considerable time—it was productive of great uneasiness—and it was deliberately placed in the position which it held, in order to answer certain purposes, which do not require to be more particularly specified. But this will be a lesson to you, exhorting you to be upon your guard, and to take care that you do not implicitly rely upon representations which you cannot yourselves confirm."

Appended to the foregoing are some clinical remarks, by Mr. L. on a case of gonorrhœal ophthalmia in a female. The case itself is not of much moment, nor the observations of much importance. Mr. Lawrence is made to remark, that he has never before seen acute gonorrhœal ophthalmia in a female. In his work on Gonorrhœal Ophthalmia and Syphilitic Iritis, he gives the particulars of a case of this description, communicated to him by Mr. Macilwain, and as there can be no good reason why males should suffer exclusively from the disease, and, further, as these cases alone prove that females do suffer occasionally, we conceive that the paucity of such instances in Mr. Lawrence's practice, must be in some measure the result of accident. Still, it cannot be denied that men are attacked in a much larger proportion than women. In our review, or rather analysis of the work of Mr. Lawrence, to which we have already alluded, we remarked the almost singular reluctance evinced by Mr. Lawrence to the employment of astringents or stimulants in the more acute affections of the eye. It appears from the clinical lecture before us, that this reluctance is a little diminished. It is hard to forego cherished opinions, and abandon practices consecrated by time and theory. Yet those who will not only think, but experiment for themselves, and those who will watch without prejudice the bold experiments of others, will probably venture farther than Mr. Lawrence has yet done. We had very lately an opportunity of witnessing a case of gonorrhœal ophthalmia, treated by Mr. Guthrie at the Ophthalmic Infirmary, which he has erected, with infinite credit to himself and advantage to the poor, in the neighbourhood of Charing Cross. The inflammation of the conjunctiva was treated by means of the ointment of the nitrate of silver, assisted by moderate local depletion. The eye was perfectly saved.

### II. Chimney Sweeper's Cancer.

Mr. Earle has made some observations on this disease, which are not undeserving of attention. The subject of the case was a patient named Bennett, æt. 28, who had an affection of the inguinal glands, apparently produced by soot, and analogous to chimney sweeper's cancer. There was a deep chasm in the groin, surrounded by ulcerated edges, which seemed disposed to throw out a fungous growth, and exhaled the ammoniacal odour, which Mr. E. considers as characteristic of the disease as an acid odour is of rheumatism, and a gangrenous one of phagedæna. On investigation, the patient admitted that, about 15 years previously, he had been employed in carting soot, and, at times, had warty excrescences on the scrotum and groin; at last the inguinal gland became affected. Mr. Earle has paid some attention to the subject, and presents the following as the results of his enquiries.

"The chimney sweeper's cancer has been classed amongst the number of malignant diseases, and is thought to be at first caused by a peculiarly stimulating property contained in soot. It was well known that a great deal of ammonia was contained in soot, but what it was that caused the disease in question, had not been as yet ascertained. However, it was well known to be peculiar to those countries where coals were used as fuel. This disease was described by Pott, who had published the only accurate account on the subject. He (Mr. Earle) having formerly attended a number of stations occupied by the sweeps, had been enabled to collect a mass of cases of this disease, the results of which were confirmed by subsequent experience. As that inquiry had led him to form some exceptions to the rules laid down by Pott, it was the more important to state them for the information of the pupils. The disease in question was invariably produced by soot, applied to the rugæ of the skin and scrotum; it commences with warty excrescences, which may remain stationary for years, and depending upon various circumstances. The patient in the present case was attacked gradually till six years ago. After some time the disease reaches the skin, throwing out a peculiar growth with a scirrhous hardness, and a peculiar odour very different from that which proceeds from those warts that are sometimes found on males and females from other causes. This growth increases by degrees, and ultimately involves the parts contiguous. According to Mr. Pott, the disease always commenced on the lower part of the scrotum; but this was not invariably the case, as the following remarkable instance would prove: A few years since, a gardener, shortly after strewing some soot over a garden, to destroy slugs, observed an extensive growth of warts make its appearance upon his wrists. Amputation was found requisite to insure the patient's recovery. This disease will sometimes attack the common integument of the cheek, and he himself had seen many instances of this kind in chimney sweepers. The disease, when it attacked the scrotum generally, advanced to the adjoining parts, spreading to the perineum and boundaries of the anus, not unfrequently affecting the testicle; ulceration and sloughing sometimes take place, and spread to the body of the testicle. It would appear from various observations, that its progress in glandular parts was different from that which takes place in the common integument. In the present case, a large chasm formed in the groin, and was making its way into the cavity of the abdomen, the edges throwing out that peculiar secretion which is so characteristic of this affection. The disease always spreads to the contiguous parts, clearly proving that it did not commonly proceed in the course of the absorbents. He (Mr. Earle) was acquainted with a case in which a bubo made its appearance: the bubo suppurated, and assumed a character similar to the primary affection. The individuals attacked by this disease were generally of a pallid hue, with wan and leaden countenances; their general health was greatly affected; but the circumstance which particularly deserved their attention was that peculiar secretion and ammoniacal smell which, if once recognized, could never be mistaken. Indeed, the odour in such cases formed as certain a diagnosis, as the smell in various other diseases, such as rheumatism, phagedena, &c. It would be of great use to recollect these distinguishing signs, as they lend much assistance to the surgeon. The infrequency of this disease would be explained, by its seldom attacking persons under thirty years of age; very few cases occurred between twenty and thirty. Children were not liable to this affection; and it was some consolation to humane individuals to be aware of the fact, as it was generally supposed that the children bound to this trade, were doomed to a life of suffering in every respect. The man now in the hospital, had never been apprenticed to the trade, and was not attacked with the disease for a considerable number of years. From a long course of observation, he (Mr. Earle) considered that there were strong grounds for supposing that a constitutional predisposition was required for the development of this disease, an idea which was materially corroborated by the fact of children not being liable to its ravages. Most of the cases which he had observed, were in persons between the ages of thirty and forty; and it was often twenty years before those individuals were affected, as was fully proved in the case of the patient now in the hospital. It was also a singular fact, that the disease shewed itself in particular families; as he had known two or three generations which had fallen victims to its effects, and all about the same period of life. Indeed, there could be no doubt that a certain state of constitution gave rise to the disease.

With respect to the mode of treatment, he had no hesitation in saying, that, as far as he knew, no topical or internal remedy could be of use; the scalpel was the only resource, and it might be relied upon with confidence, provided that care was taken to remove the entire of the diseased mass. When the glands in the groin were affected, he pursued the same practice; even when the testicle was attacked with disease, it would be right to give the patient a chance by removing the affected testis, provided the spermatic cord were sound. A case, illustrative of the success of this practice, occurred last July twelve months, in a patient who came to the hospital in a hopeless state. The inguinal glands were enlarged; there was a local affection occupying the whole of the scrotum; the testicles were involved in the disease, so that it was necessary to remove both; the entire scrotum was removed, with both testes, and it was even judged prudent to dissect away a considerable portion of the corpus spongiosum of the urethra. Notwithstanding this extensive excision, the patient is now alive, and perfectly well; and the disease in the groin is subdued. The man ran a great risk, as considerable inflammation of the integuments supervened; there were large sheets of sloughing of the integuments, and at one time he had a slight attack of trismus; the wound sloughed in the perinæum, and allowed an exit to the urine in that part, but it subsequently closed, and the man now makes water in the ordinary way. This case afforded a proof of the entire locality of the disease. In another instance, the disease was so completely local, that it was only necessary to remove part of the tunica albuginea. As the result of his experience, he should in all cases recommend the extirpation of the diseased part, and to some extent beyond its influence. But he would at the same time require the patient to abandon his business, as a return to it would be almost certain to bring on a return of the disease. From the rapidity with which this disease proceeded, when once established, no time should be lost, and the operation should be extensively performed. He was sorry to add, that the present case was beyond the power of the knife, for the ravages of the disease in the groin were of so malignant a description, and they spread so rapidly to the cavity of the abdomen, that he could only look forward to a fatal termination.

He had heard of various remedies employed for this disease, and particularly *iodine*, lately tried in a case at St. George's Hospital, but he understood it produced no good effect. In his opinion, time would only be lost by using such remedies, as the only certain plan was the immediate extirpation with the scalpel."

We may take the liberty of offering a comment or two on the foregoing valuable observations. There can be no question that the disease is not confined to the scrotum, as one of its names, "cancer scroti," would lead the inexperienced practitioner to believe. The cases mentioned by Mr. E. are decisive on this point; and we may add that we have twice seen it in the groin, unaccompanied with any scrotal implication. Mr. Earle pronounces that children are not liable to it. This is not the fact. A boy was lately a patient in St. George's Hospital, with every appearance of this complaint in his left groin. He was a chimney-sweeper, and the warty ulcerated growth had most of the characters of this form of cancer. There was also considerable enlargement of the deep iliac glands. The boy took iodine, under the direction of Mr. Brodie, the ulcerations healed, the warty growths disappeared, and the enlargement of the glands was diminished. Whether the boy continues well we do not know. We suppose this is the case to which Mr. Earle alludes, at the conclusion of his lecture; how erroneously, our readers may perceive! This is a slight sample of the looseness with which medical matters are too frequently disposed of. We must then conclude that "the fact of children not being liable to the disease," is probably not a fact, and, inferences drawn from it are possibly as fallacious as their foundation.

### III. Case of Strangulated Hernia—Intestine wounded in the Operation.

H. Taylor, æt. 45, was admitted at 5, a. m. of the 22d January, with an inguinal hernia so tense as to preclude all hope of reduction by the taxis. "The *usual* means, *of course*, were employed for that purpose; *the warm-bath, venesection, ice, and tobacco glysters.*" The operation, on the failure of these usual means, was proposed, but rejected, until half-past 12 o'clock, when pain having commenced about the lower part of the abdomen the patient consented to its performance.

"After the sac had been cut into, the convolution of small intestine which presented, might measure from five to seven inches. Its appearance was generally of a favourable nature, except that it was somewhat preternaturally reddened. In consequence of the employment of a very small and shallow director, which proved quite insufficient for the necessary degree of protection to the surrounding parts, the division of the stricture was attended by a wound of the intestine. A yellowish watery fluid flowed out, resembling the morbid secretion from the serous membrane of the abdomen, and might be easily accounted for by referring to the state of irritation produced by the hernia; but the secretion, as afterwards appeared, came from the mucous membrane of the intestines. A ligature was tied round the wound, and the intestine was returned to the abdomen. After its disappearance, the operator seemed surprized at finding *behind* the situation of the protruded intestine, a large mass of omentum, which was attached by very firm adhesions to the posterior surface of the sac. The ordinary state of entero-epiplocele is—the omentum anterior to the bowel; and Mr. Lawrence had no recollection of having met with such a case as this before. A considerable portion of the omentum was removed; and the vessels being very large, gave out a copious discharge of blood, and a considerable number were tied. The patient was then put to bed, and allowed to remain quiet for two or three hours, after which he took compound colocynth pill and sulphate of magnesia, at repeated intervals; but without effect. The pain in the abdomen increased in the course of the evening; leeches were applied in abundance, and purgatives repeated, but no motion, except the watery secretion already mentioned, followed. Next morning he complained of great weakness; the pulse was low, and the surface of the body rather cold. Cordials and stimulants were employed without effect, and the patient died about one o'clock in the afternoon of the 23d, just 24 hours after the operation."

*Sectio Cadaveris.* Peritoneum generally healthy, but reddened over the convolutions of the intestine on the side of the hernia. Omentum seen stretching downwards to the abdominal ring, where it was firmly secured by old adhesions; the intestines were compressed and indented by it in this course. The colon at the point of its attachment to the omentum was constricted, and so reduced in diameter as to oppose an impediment to the passage of the fæcal matter. The convolution of intestine, which had been protruded into the hernial sac was somewhat inflamed, presenting spots of vascularity and ecchymosis, and being agglutinated to another portion of intestine. The wounded part and every portion of the ligature was completely concealed from sight by a copious effusion of coagulable lymph ; rendering it probable that, had life been preserved, the ligature would have ultimately been discharged into the cavity of the intestine. More disease was discovered in the brain than had been anticipated. The vessels of the dura mater were numerous and dilated ; the arachnoid membrane thick and opaque and giving a milky appearance to the surface of the brain ; whilst a copious serous effusion had also taken place into the pia mater. Upwards of three ounces of very pellucid fluid were found in the right lateral ventricle ; the right corpus striatum had lost its colour, was brown and flaccid, as were the adjacent parts, which had been produced, no doubt, by a former effusion of blood into the part. The patient, we should mention, had had some paralytic affection of the left arm.

We must again take the opportunity of making a remark or two. The injury of the intestine in the operation scarcely deserves one, for those who see most of, and we may add those who have most practical acquaintance with, operations, will ever be found the most lenient to accidents of this description. It is your pert coxcomb who never fledged aught but lancet on human patient, perhaps not that, or your malignant knave, who loves to scarify reputations in the dark, it is these and these only who blazon failures as deeds of ignorance and imbecility. We trust, for the credit of human nature, and particularly of our own profession, that such practices and the taste for their encouragement are alike passing away. Let the press be as free as freedom herself, but the good cause is injured, the press itself is injured, when that liberty is abused, and lewd licentiousness unrestrained and unrestrainable prevails. Our readers know the liberality of our sentiments too well, to suspect that we could wish for aught but its perfect triumph.

We cannot forbear noticing the passage in the body of the case which we have placed in Italics. Does the reporter mean to assert that the means *of course* in the treatment of irreducible hernia, are "warm-bath, venesection, ice, *and* tobacco glysters." God help the patients of any surgeon or of all surgeons if they were so ! They certainly are not means of course, they require discrimination in their selection, in their application, and so far from their being ordinary measures, we do maintain that in very few cases indeed can this string of powerful means be employed. In the hernia-patients whom we have seen, one was frequently, two generally sufficient, three could seldom be ventured on, the whole we might almost say never. In short, it would be neither more nor less than extremely bad practice to resort to all these measures, or even to the majority of them, previous to operation, in the greater number of cases.

We do not altogether participate in the astonishment evinced by the reporter at the state of the arachnoid, and corpus striatum. The condition of the former is very frequently witnessed where post mortem examinations are carefully and commonly performed, unaccompanied, as here, with any prominent symptoms during life. In a former number of this Journal we related some cases of this kind, and drew particular attention to the fact. The relics of an ancient extravasation in the corpus striatum, are also one of the most ordinary cerebral alterations discovered in pathological investigations. The reporter will probably grow older and wonder less. One word more and we have done. Mr. Ashwell, we are informed, is curing, nay, has six times cured "scirrhus uteri," by the hydriodate of potass : two or three grains of which, with six of extract of hyosciamus, are introduced into the rectum every night. Dr. Elliotson, on the contrary, proclaims that hydriodate of potass may be given in drachm doses, and is merely a diuretic. Now we are moderate persons. We should not, therefore, give drachm doses of the hydriodate, until we had felt our way with it, and if in less doses it did no good we should take care to ascertain that in greater it would do no harm. There are stories abroad respecting the effects of iodine on the testicles and mammæ, that should make people cautious. Again, we would scarcely be so uncourteous as to declare that we do not believe that Mr. Ashwell has cured six cases of "scirrhus uteri" by the hydriodate, and yet in point of fact we do—not believe it. We have seen that game played, and we have played it ourselves, but alas! after much trouble and some time there was the scirrhous uterus, never better, and often worse. No. We must have a new specific. This new journal is in truth consolatory. Dr. Seymour, it tells us, is employing iodine in diseases of the ovaries with manifest advantage. "The diminution of ovarian tumours is most remarkable." Be it so. But think Dr. Seymour, of your patient in the Queen's ward — think of Dr. Anthony Todd Thompson. This latter most able and truly estimable physician cured a case of ovarian disease by iodine at the University Dispensary. The poor woman unfortunately died of it at St. George's, and unless we are mistaken, a portion of her mortal remains is now preserved by the care of the curator and at the cost of the hospital. Enthusiasm is a very good thing in young journals, as in young people; but caution for us, who may now be said to be grey in our vocation!

---

V.

HISTORY OF THE CHRONIC PHLEGMASIÆ, OR INFLAMMATIONS. By M. BROUSSAIS; translated from the 4th French edition by Drs. HAYS and GRIFFITH, of Philadelphia. Longman and Co. London. 2 vols. 8vo. 1831.

THE work, here presented in an English dress, is unquestionably the best, as it was the first which came from the pen of that extraordinary man—BROUSSAIS. The other productions of this author have been handled severely by various critics—and we ourselves have taken the liberty to offer some commentaries both on the doctrines and practice of the distinguished founder of a large sect on both continents of the world; but the history of the chronic inflammations has always and every where been considered "a model of knowledge and originality in medicine." The work in question was originally published in 1808, and required eight years to arrive at a second edition; but since that period it has come to a fourth edition, and is to be found in the libraries of most physicians who study French writers. There is a large proportion of medical practitioners, however, both in this country and in America, who either do not, or cannot peruse the original work of Broussais, and to these we strongly recommend the present translation. We fully agree with the transatlantic editors in the following encomium.

"The History of Chronic Phlegmasiæ is unquestionably the *chef d'œuvre* of the most remarkable medical writer of the present day. For pure observation, just and discriminating views, and rigid adherence to the spirit of the inductive philosophy, few medical works can compare with it. No where else in medical literature is there to be found, a more copious array of facts carefully observed—morbid alterations met with after death connected with the antecedent symptoms—and the modifying effects of remedies minutely pointed out. Assailed as the other productions of this author have been with unprecedented virulence and acrimony of criticism, the present has extorted praise even from the most violent opponents of the physiological school."

The translators had a difficult task to perform, since the style of M. Broussais is remarkable, not only for force and boldness, but too often for harshness and abruptness. The translators, therefore, acted wisely in giving up the attempt at smooth and polished style, in this enterprize, preferring rather to render the descriptions and tenets of the author as accurately as possible. We think that Drs. Hays and Griffith have acquitted themselves very fairly in this translation, and the following passage will at once prove a sample of the work, and exhibit some useful hints to the medical practitioner.

"It is known to all physicians who walk hospitals, that there are met with in these establishments a number of pale, emaciated patients, who are daily losing their strength, and advancing slowly towards the tomb, with a more or less distinctly characterized hectic fever, and sometimes without any appreciable febrile action.

The reflections required for the composition of my work on hectic fever, had fixed my attention upon these unfortunate and too long neglected individuals; and as soon as I entered into the service of military hospitals, I resolved to devote myself to the particular study of chronic diseases.

When I sought for a guide among the most illustrious authors, and those to whom medicine confessedly owes its greatest advances, I found only confusion—every thing was conjectural. I immediately perceived that facts were wanting, although pathological anatomy had already been much enriched by Bonetus, Morgagni, Lieutaud, and by the investigations made after the example of the celebrated Bichat, by distinguished physicians of the school of Paris.

The deficiency was not in the number of post mortem examinations, but in the individual histories of cases, and in the connexion of the symptoms. At the present advanced state of the science, a good treatise on chronic diseases can no longer be a compilation, and still less a collection of observations, registered by different reporters, and collated by a person who has not seen the patients.

It was necessary for the presentation of this matter in a luminous manner, that a hospital physician should himself undertake the laborious task of collecting and digesting the histories of diseases. That these histories might be complete, it was indispensable for him to watch the progress of the disease to its termination, and that he should verify it positively, by assuring himself of the continuance of the health of the patient, or by making an autopsy of his body. This labour should not be confided to students, because the art of observing is difficult, and every reporter in framing his account, is influenced by his peculiar views and principles, and interprets nature differently.

It also appears to me, that the most happily organized physician requires the whole of his faculties to examine all the symptoms of a protracted disease; I even persuade myself that it is only by dint of repeating these examinations, that he can habituate himself to the language of suffering nature—that he can ensure the rigorous and uniform progress of the medicine of observation—that he can, by rectifying the doctrine, extend it, render it more correctly appreciated, and contribute to the progress of the science of man, by perfecting the art of curing his numerous infirmities."

As this translation can now be obtained from Longman's house, we strongly recommend it to the attention of our professional brethren.

---

### VI.

Practical Observations on Cholera Asphyxia, &c. By James B. Kirk, M.D. 8vo. pp. 100. Greenock, May, 1832.

We must be very brief with pamphlets and indeed all works on cholera now. Yet it would be an injustice to the public, as well as to those zealous practitioners who have investigated the epidemic, to pass entirely unnoticed the results of their labour. Dr. Kirk appears to be a most philanthropic physician. From Greenock, he proceeded to Edinburgh, Tranent, Musselburgh, and Newcastle, studying the epidemic—and, at length, after multiplied observation, and extensive epistolary communication, coming to the conclusion that diarrhœa is *invariably* the first stage of the disease—and that, in such stage it is a very manageable complaint. That we are of the same opinion the readers of this Journal will testify. In not a single case, where we have been able to investigate the circumstances personally and carefully, have we failed to find that diarrhœa was the primary link in the chain of morbid phenomena. The great value of Dr. Kirk's pamphlet consists in the train of communications from upwards of twenty medical practitioners conversant with the epidemic, and who bear ample testimony to the truth of Dr. Kirk's position respecting diarrhœa. Among these we may note Dr. White, of Gateshead—Dr. Morson, of Newcastle—Messrs. Morson and Leighton, of the same place—Messrs. W., T. K., and J. Fife, do.—Mr. M'Intyre, do. — Mr. Tulloch, do. — Mr. Hardcastle, Mr. Frost, Mr. Clark, Mr. Greenhow, do. — together with many other experienced practitioners of the same place, and of Gateshead. From Mr. Lizars, of Edinburgh — from Mr. Meikle, of do.—from Dr. Carruthers—from Mr. Cunningham, of Tranent—Mr. Moir, of Musselburgh—Mr. Steele —Dr. Dunbar—Mr. Maccall—Mr. Sanderson — Dr. Mollison — Dr. Johnson, &c. &c. Our author has given proofs the most cogent that diarrhœa is the first stage of cholera—proofs that might stagger the most ardent sticklers for "perfect identity," between our epidemic and that which ravaged India. We shall take the following description of the first stage of the epidemic from our author.

"The patient complains of lassitude. He has frequently partial uneasiness in the region of the stomach; but this not to such a degree as to alarm him. He has frequent evacuations from the bowels—from two to a dozen times a day—not attended with much griping. His countenance is sharp and dark. He knows not of this symptom, and it is only recognizable to the eye of experience. Occasional nausea may oppress him. But this is not a very common symptom. These symptoms may continue, varying in severity, from one to ten days, before the second stage of the disorder supervenes. The evacuations at the first are generally of a dark brown or blackish hue. As the looseness continues they gradually become less and less of a natural appearance, until they assume the consistence and aspect of dirty water. Some headache, cramp of the fingers, toes, and abdomen, and almost always slight giddiness and ringing of the ears, accompany these symptoms. Sometimes an intervening two or three days of costiveness supervenes, which is followed again by the diarrhœa, and in a few hours collapse supervenes, and in general nausea and vomiting. The skilful practitioner will now give pills composed of aloes and calomel, or a pill composed of scammony, calomel, and aloes. The bowels then in general act briskly. Continue the course for three days—keep the patient warm in bed — give him mild and gentle nourishment; and, after an immense quantity of horribly offensive dejections, the patient is completely recovered, and snatched from the jaws of the dreadful fate which awaited him."

Dr. K. proposes a plan of exterminating cholera — namely, an examination daily in each town where it breaks out, of the inhabitants, in order to arrest the primary stage, by proper means. This, in truth, is the only sanatory cordon of any efficacy. In our reflections on the Board of Health in Musselburgh, and their contagious doctrines, we were rather severe. But let the reader peruse the following extract from Dr. Kirk, and say whether or not we had justice on our side.

"It was evident to me, from the eventful experience of these two days, that little was to be learned of the right treatment of this disease in Musselburgh and Tranent. The practitioners were in dismay, and knew not what hand to turn to. Their remedies were as various as they were uncertain; and I retired from the scene, where the Opprobrium Medicinæ stared me in the face, and taught me an impressive lesson of how poor a thing our boasted art is when God is against us. Illness persuaded me to go no farther: the love of my country and of my townsmen, who have so liberally supported me for half a life time, called on me to proceed to a place where the disease had long existed, where panic had subsided, and where I had heard that British energy, and British skill, and British science, had been brought to bear upon this terrible complaint. I proceeded next morning to Newcastle; and how rejoiced was I to find, after a single inspection of their hospitals, the disease managed upon a correct system, the medical attendants *calm and philosophical,* the indications of cure flowing out from premises easily to be understood; and all that was *obscure, mysterious, and empirical, in Tranent and Musselburgh,* was now dependant on rules of science easily comprehended."

Yet the practitioners of Musselburgh have designated all opponents to their pusillanimous and fanatic doctrines as ignorant and incorrigible sceptics!

Dr. K. is a non-contagionist—or, at most, a very moderate contingent contagionist. With the following extract we must close this short notice.

"Now, after all these facts, are we to pronounce the disease infectious? We have not an approach to a reason to say that it is so. The experience of India, Russia, England, and Scotland militate against it, for in all these countries the attendants of the sick have not been attacked in a larger proportion than the rest of society, unless that attendance had been in ill-ventilated places, where a malaria atmosphere was suddenly perfected; and thus are all the inhabitants of the little district exposed to disease in the same way that the people of an extended country have it, the atmosphere of which has, from inscrutable causes, become choleric. It is here that the contagionists have committed their grand error, in not distinguishing between an atmosphere imbued with disease, and the power of infecting directly by touch or close communication.

It will be seen by these observations that the doctrine I hold is that of contingent contagion; for, undoubtedly, facts most clearly evince, that there are circumstances in which the atmosphere of places becomes so contaminated that they directly cause the disease. But this exists in Cholera to a much less extent than in typhus fever and most of our contagious diseases,—so little, indeed, that we may look on this disease as one which may be considered non-contagious, except under the combined influences of many predisposing causes in the person attacked, and the effect of an atmosphere vitiated by the emanations of accumulated disease."

The appendix of communications from various practitioners, many of them distinguished ones, is highly deserving of perusal, and we recommend Dr. Kirk's modest pamphlet, in the strongest terms, to our professional brethren.

